# Planktonic Microbes in the Gulf of Maine Area

**DOI:** 10.1371/journal.pone.0020981

**Published:** 2011-06-15

**Authors:** William K. W. Li, Robert A. Andersen, Dian J. Gifford, Lewis S. Incze, Jennifer L. Martin, Cynthia H. Pilskaln, Juliette N. Rooney-Varga, Michael E. Sieracki, William H. Wilson, Nicholas H. Wolff

**Affiliations:** 1 Fisheries and Oceans Canada, Bedford Institute of Oceanography, Dartmouth, Nova Scotia, Canada; 2 Bigelow Laboratory for Ocean Sciences, West Boothbay Harbor, Maine, United States of America; 3 Graduate School of Oceanography, University of Rhode Island, Narragansett, Rhode Island, United States of America; 4 Aquatic Systems Group, University of Southern Maine, Portland, Maine, United States of America; 5 Fisheries and Oceans Canada, Biological Station, St. Andrews, New Brunswick, Canada; 6 School for Marine Science and Technology, University of Massachusetts, North Dartmouth, Massachusetts, United States of America; 7 Department of Biological Sciences, University of Massachusetts Lowell, Lowell, Massachusetts, United States of America; Argonne National Laboratory, United States of America

## Abstract

In the Gulf of Maine area (GoMA), as elsewhere in the ocean, the organisms of greatest numerical abundance are microbes. Viruses in GoMA are largely cyanophages and bacteriophages, including podoviruses which lack tails. There is also evidence of Mimivirus and Chlorovirus in the metagenome. Bacteria in GoMA comprise the dominant SAR11 phylotype cluster, and other abundant phylotypes such as SAR86-like cluster, SAR116-like cluster, *Roseobacter,* Rhodospirillaceae, Acidomicrobidae, Flavobacteriales, *Cytophaga*, and unclassified Alphaproteobacteria and Gammaproteobacteria clusters. Bacterial epibionts of the dinoflagellate *Alexandrium fundyense* include Rhodobacteraceae, Flavobacteriaceae, *Cytophaga* spp., *Sulfitobacter* spp., *Sphingomonas* spp., and unclassified Bacteroidetes. Phototrophic prokaryotes in GoMA include cyanobacteria that contain chlorophyll (mainly *Synechococcus*), aerobic anoxygenic phototrophs that contain bacteriochlorophyll, and bacteria that contain proteorhodopsin. Eukaryotic microalgae in GoMA include Bacillariophyceae, Dinophyceae, Prymnesiophyceae, Prasinophyceae, Trebouxiophyceae, Cryptophyceae, Dictyochophyceae, Chrysophyceae, Eustigmatophyceae, Pelagophyceae, Synurophyceae, and Xanthophyceae. There are no records of Bolidophyceae, Aurearenophyceae, Raphidophyceae, and Synchromophyceae in GoMA. In total, there are records for 665 names and 229 genera of microalgae. Heterotrophic eukaryotic protists in GoMA include Dinophyceae, Alveolata, Apicomplexa, amoeboid organisms, Labrynthulida, and heterotrophic marine stramenopiles (MAST). Ciliates include *Strombidium, Lohmaniella, Tontonia, Strobilidium, Strombidinopsis* and the mixotrophs *Laboea strobila* and *Myrionecta rubrum* (ex *Mesodinium rubra*). An inventory of selected microbial groups in each of 14 physiographic regions in GoMA is made by combining information on the depth-dependent variation of cell density and the depth-dependent variation of water volume. Across the entire GoMA, an estimate for the minimum abundance of cell-based microbes is 1.7×10^25^ organisms. By one account, this number of microbes implies a richness of 10^5^ to 10^6^ taxa in the entire water volume of GoMA. Morphological diversity in microplankton is well-described but the true extent of taxonomic diversity, especially in the femtoplankton, picoplankton and nanoplankton – whether autotrophic, heterotrophic, or mixotrophic, is unknown.

## Introduction

The Census of Marine Life is a global assessment of the diversity, distribution and abundance of life in the ocean [1]. Hemispheric-scale reviews have assessed biodiversity in pan-Canadian [2] and pan-American waters [3]. However, for the purpose of ecosystem level considerations, there is a need to further downscale the Census to a regional level. One such region is the Gulf of Maine Area (GoMA), selected by the Census program because it is a place of significant economic, cultural, political, scientific, and educational interest. Although this distinct large marine ecosystem has a long history of plankton studies [4], few, if any, integrated assessments of this system [5], [6] have explicitly considered the diversity of planktonic microbes, except for recognition of the phytoplankton as primary producers. More than 20 years ago, a review of marine microbiology for Georges Bank (part of GoMA) [7] stood at the cusp of the paradigm shift leading to the contemporary era of the microbial loop. The construct of a linear food chain from diatoms and dinoflagellates to copepods to fish was replaced by a complex non-linear food web that recognizes the diversity of form and function in unicellular organisms. Here, we review the current state of knowledge for planktonic microbes in GoMA as a contribution towards an integrated approach to the understanding and stewardship of this ecosystem [8].

## Gulf of Maine Area

**Figure 1:**
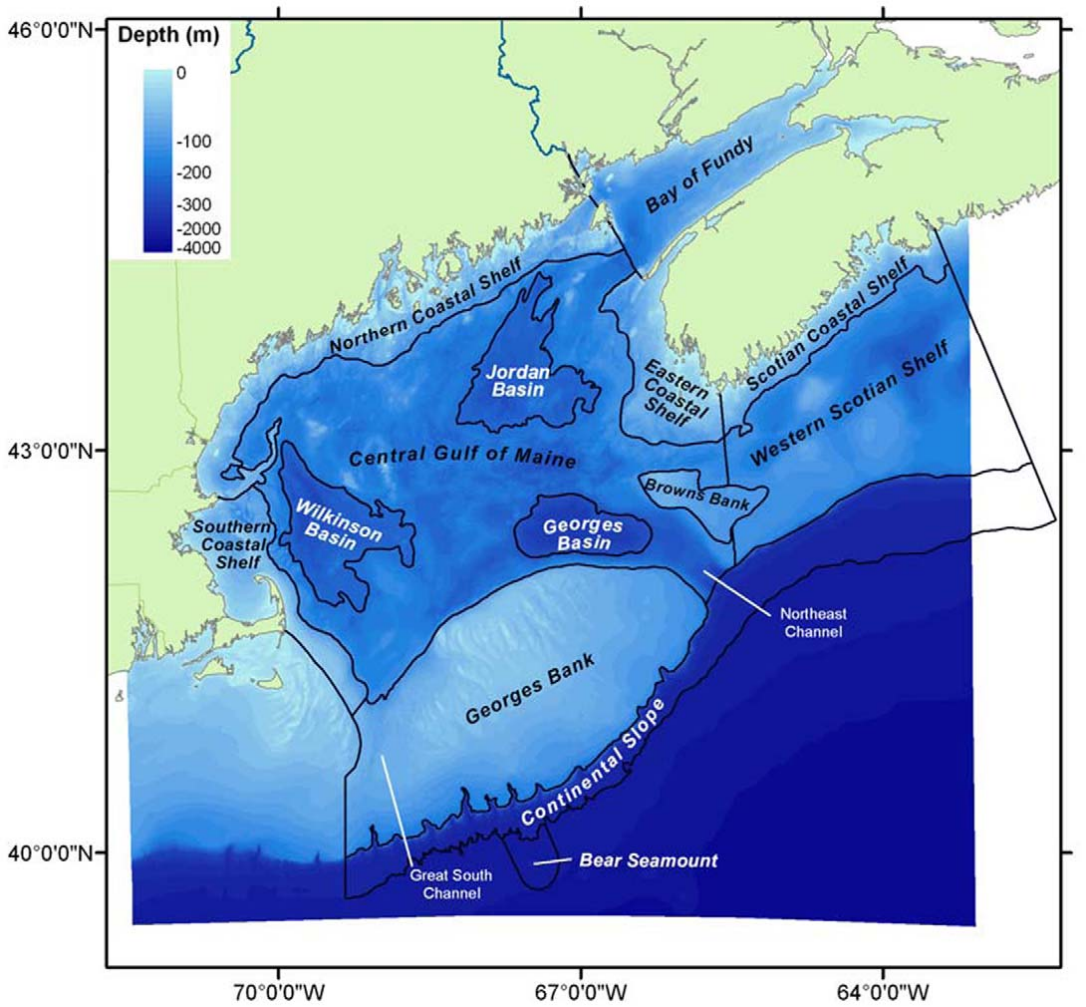
Gulf of Maine Area map. Fourteen physiographic regions are defined for this study (Text S1). The shaded bathymetry is from United States Geological Survey 15 arc sec data used in the hypsometric calculations. High-resolution data were not available for the portion (2%) of the study area that is not shaded.

The Gulf of Maine area is bordered by the New England coastline of the United States (Maine, New Hampshire and Massachusetts) and the eastern maritime provinces of Canada (New Brunswick and Nova Scotia). The eastern boundary is delimited by a line normal to the coast of Nova Scotia extending from Halifax, the southern boundary by the 2000 m isobath on the continental slope, and the western boundary by Nantucket Shoals and the western side of the Great South Channel (Fig. 1). GoMA can be partitioned by physiography into 14 regions that comprise the coastal shelves (Scotian Coastal Shelf, Eastern Coastal Shelf, Northern Coastal Shelf, Southern Coastal Shelf), the Bay of Fundy, the open shelves (Western Scotian Shelf, the central Gulf of Maine proper), offshore banks (Georges Bank, Browns Bank), major basins (Georges Basin, Jordan Basin, Wilkinson Basin), and deep waters (continental slope, Bear Seamount). The Western Scotian Shelf also has a bank and basin topography, with two basins (LaHave, Emerald) deeper than 250 m. In total, these regions account for 0.3% of the surface area and 0.01% of the volume of the North Atlantic Ocean (Text S1).

In a larger physical context, GoMA may be considered one of six components of the northeastern North American coastal ocean system, formed by the Labrador Shelf, the Newfoundland Shelf, the Gulf of St. Lawrence, and the Scotian Shelf to the north, and the Mid Atlantic Bight to the south [9]. These components largely correspond to an ecological sub-partition of the Northwest Atlantic Shelves Province (NWCS) of the Atlantic coastal biome [10] in which GoMA lies on the northeast shelf midway between Cabot Strait and Cape Hatteras. A comprehensive oceanographic description of the northwest Atlantic continental shelf including the Gulf of Maine [11] underscores the importance of the physical setting (physiography, circulation and water masses, North Atlantic Oscillation, frontal features, the Gulf Steam) to ecosystem considerations. Thus, for metazooplankton and pelagic nekton, it is evident that factors such as bathymetry, proximity of the coast, advection, and shelf-slope mixing shape the patterns of biological diversity [12].

## Viruses

### Genomic diversity

The Global Ocean Survey (GOS) collected virus fraction concentrates by tangential flow filtration at all stations, including those in GoMA [13], but the analysis of these virus fractions apparently has yet to be reported. However, virus sequences in the microbial fraction (0.1 µm–0.8 µm) for GoMA GOS samples GS02 - GS07 [14] have already shed light on the larger free-viruses (which are not associated with hosts) and any viruses that were infecting microbial hosts at the time of sampling. Sequence data from these GOS microbial fractions revealed that viruses are clearly an abundant component representing approximately 3% of the total predicted proteins [14]. The predominant virus sequences throughout the GOS subset originated largely from tailed bacteriophages. However, analysis of the spatial distribution of virus sequences revealed that GoMA has a higher proportion of sequences from podoviruses, a family of bacteriophages that lack tails. An effort to map the distribution of genes from a *Prochlorochoccus*-specific cyanophage genome (P-SSM4 [15] across the GOS subset, showed no hits in GoMA [14]. This is not surprising, since *Prochlorococcus* spp. have not been reported in the Gulf of Maine, as expected for shelf waters north of the Gulf Stream.

In a taxonomic analysis of large DNA viruses in the GOS metagenome, using the viral DNA polymerase gene as taxonomic marker, a high proportion of Chlorovirus and Mimivirus homologues were observed in GoMA [16]. Observations of Chloroviruses are worth noting since they are likely of freshwater origin. Their green alga hosts are small, unicellular, non-motile, asexual green algae with a global distribution. To date, the only described chloroviruses infect symbiotic *Chlorella*, often referred to as zoochlorellae, such as those associated with the protozoan *Paramecium bursaria* or the coelenterate *Hydrozoa viridis* [17], [18], [19]. Mimivirus is the largest known virus at 1.2-Mega base-pairs [20]. Taxonomically, there is only a single representative in the family Mimiviridae though it is likely that Mimivirus homologues discovered in the GOS database are related to algal viruses [21].

### Viral isolates

Viruses are obligate parasites and require a susceptible host as a starting point for isolation studies. For microbial hosts, viruses are typically isolated by adding filtered seawater (most viruses pass through a 0.2 µm filter) to a culture of host cells or by enriching the filtered seawater with nutrients prior to adding host cells of choice. Clonal purification of viruses can then be conducted by plaque assay using standard microbiological techniques. GoMA viral isolates have been studied in this way. Favourable physicochemical conditions make GoMA an ideal location for frequent mesoscale blooms of the coccolithophore *Emiliania huxleyi* that usually occur during summer stratification, and even in non-bloom years calcifying species make up a significant part of the phytoplankton community [22]. Three DNA-containing viruses that infect *E. huxleyi* (EhVs) have been isolated in GoMA [23]. The EhVs were ether-resistant isolates that possessed icosahedral symmetry and were 130–160 nm in diameter. All isolates caused complete lysis of host cultures within four days, produced large plaques on host lawns in agarose, and were highly stable at −72°C. Although no molecular characterization was conducted, these viruses are morphologically similar to coccolithoviruses, a virus genus from the family Phycodnaviridae [24], [25], a group of large double stranded DNA viruses that are known to infect *E. huxleyi* and other algae. Of the six Phycodnaviridae genera, the prasinoviruses and viruses of photosynthetic picoeukaryotes are the least represented in the literature, despite the globcal and ecological importance of their hosts. Viruses for these algal groups have been readily isolated elsewhere [26], [27]; they may be expected to be prevalent in GoMA but to our knowledge no similar isolation has been performed in these waters.

A range of marine bacteriophages and cyanophages (viruses that infect bacteria or cyanobacteria) were isolated from West Boothbay Harbor or the central Gulf of Maine in different studies between 2000–2007 for use in determining their scattering properties in seawater [28], [29]. Only some very basic characterization was conducted on these phages, focusing on their morphology, size and speed of culture lysis, all components necessary for interpreting light scattering properties. Cyanophages and bacteriophages are known to be ubiquitous and abundant in the ocean [30]. It is probable they are equally abundant in GoMA and would therefore constitute the majority (95%–99%) of all viruses in this region.

## Prokaryotes

### Bacteria

Several significant molecular studies of bacterial diversity have been conducted in GoMA in recent years. These include the GOS Expedition metagenomics project [13], a study investigating links between bacterial 16S rRNA gene profiles and phytoplankton community structure in the Bay of Fundy [31], a survey of bacterial associates of cultivated phytoplankton isolated from GoMA [32], and an analysis of bacteria associated with the major toxic dinoflagellate in GoMA, *Alexandrium fundyense* [33], [34]. Since exhaustive surveys of bacterial diversity are not feasible in a system as large and heterogeneous as GoMA, and because different sites, seasons, depths, size fractions, and molecular approaches were used in various studies, there is no consensus list of clades that are present or even abundant in GoMA. In addition, most studies to date have focused on near-surface depths and, to our knowledge, there is only preliminary information about bacterial diversity in deep water habitats, such as the benthic nepheloid layer [35]. Our intention here is to provide an overview of patterns of abundant clades that appear to be associated with particular microbial habitats within the GoMA. More detailed sequence and taxonomic data from GoMA microbial communities is available via both CAMERA [36] and MICROBIS (http://icomm.mbl.edu/microbis/) databases.

Using a shotgun sequencing approach, Rusch et al. [13] avoided the potential biases in PCR-based amplification of 16S rRNA genes [37]. Their metagenomic analyses focused on the 0.1–0.8 µm size fraction of near-surface seawater. While the volume of sequence data generated in their study was vast, the number of samples was small, so it is difficult to draw statistically robust conclusions about phylotypes that are particular to the GoMA. Their results show that members of the SAR11 cluster (Candidatus *Pelagibacter*) comprised the dominant phylotype among GoMA sequences, with other highly abundant phylotypes including the SAR86-like cluster, SAR116-like cluster, *Roseobacter,* Rhodospirillaceae, Acidomicrobidae, Flavobacteriales, *Cytophaga*, and unclassified Alphaproteobacteria and Gammaproteobacteria clusters. Members of the SAR11 cluster were ubiquitously dominant across their sampling sites (from the northern GoMA to the tropical western Atlantic) and SAR86-like and SAR116-like clusters were common (but not abundant) across GoMA and other sample types. In contrast, phylotypes that appeared more commonly in GoMA samples than in other GOS sites included two *Roseobacter* phylotypes (RCA and type b), Acidomicrobidae, Flavobacteriales, Comamonadaceae, Gammaproteobacteria type b, and Alphaproteobacteria type c.

Other studies have also identified the prevalence of the *Roseobacter* clade in GoMA. For example, *Roseobacter* phylotypes were found to be dominant members of both the free-living (0.22–5.0 µm size fraction) and the particle-associated (5–100 µm size fraction) bacterial communities in a PCR-DGGE based seasonal (February to September) analysis of near-surface seawater samples in the Bay of Fundy [31]. In addition, *Roseobacter* phylotypes were found to be prevalent among bacteria associated with diverse phytoplankton isolated from the GoMA [32]. These findings support results from other coastal environments such as in the southeastern USA [38] that have suggested *Roseobacter* phylotypes may account for 20% of the total bacterial community in coastal habitats. The fact that *Roseobacter* appears prevalent in both free-living and phytoplankton-associated habitats is also indicative of their metabolic versatility [39].

Several studies focusing on bacterial associates of the major toxic microalga in GoMA, *A. fundyense*, have revealed the importance of several other clades in GoMA microbial communities. In particular, members of the Gammaproteobacteria such as *Alteromonas* spp. and *Pseudoaltermonas* spp. were found to be dominant members of bacterial assemblages that stimulated *A. fundyense* growth [33]. These genera were also detected in direct molecular analyses of bacterial epibionts of natural *A. fundyense* populations captured from seawater samples using an immunomagnetic bead separation method [34]. This latter study also found many other Gammaproteobacteria, including phylotypes that fell within the Chromatiales, Pseudomonadaceae, Oceanospirillaceae, Colwelliaceae, and Vibrionaceae and the genera *Halomonas, Psychrobacter* to be dominant members of the *Alexandrium* epibiont community [34]. Major clades that were found to be *Alexandrium* epibionts and that were identified by Hasegawa et al. and others to be abundant in the GoMA in general included Rhodobacteraceae, Flavobacteriaceae, *Cytophaga* spp., *Sulfitobacter* spp., *Sphingomonas* spp., and unclassified Bacteroidetes [13], [31], [32], [34].

The identification of predominant microbial taxa with specific metabolic capabilities is now possible using high-speed fluorescence-activated cell sorting, whole-genome multiple displacement amplification, and subsequent PCR screening. The proof of concept for this innovation was undertaken using GoMA bacterioplankton [40]. A pilot library of 11 single amplified genomes was constructed from five flavobacteria, one sphingobacterium, four alphaproteobacteria, and one gammaproteobacterium. This technique allows a precise match of phylogenetically informative genes (such as 16S rRNA) with functional genes (such as proteorhodopsin) in single cells. In addition, through bioinformatics, the Gulf of Maine results can be used to interpret survey data from elsewhere in the Atlantic Ocean [41]. The combination of single cell genomics and metagenomics is therefore a powerful new way to study the genome content, metabolic adaptations, and biogeography of numerically significant, uncultured marine microbes.

### Cyanobacteria

The diversity of cyanobacteria in GoMA encompasses 22 taxonomic names belonging to 13 genera, which are *Anabaena, Chroococcus, Gloeocapsa, Lyngbya, Merismopedia, Microcystis, Oscillatoria, Phormidium, Planktothrix, Rhabdoderma, Spirulina, Synechococcus,* and *Trichodesmium* (Table 1). The records include some forms generally considered to be freshwater but they were found in coastal, estuarine, and tidal river habitats that are considered part of the Gulf of Maine. The dominant photo-oxygenic prokaryote in GoMA is Synechococcus, a complex of organisms with similar morphology. These picoplanktonic cyanobacteria are common in many temperate coastal regions and their annual cycle of abundance at various locations in GoMA is well-established [42], [43], generally showing a maximum around the autumn equinox [44], [45].

**Table 1:** Cyanobacteria in the Gulf of Maine area.

Super Group	Division	Class	Scientific Name
Eubacteria	Cyanophyta	Cyanophyceae	*Anabaena sp.*
Eubacteria	Cyanophyta	Cyanophyceae	*Chroococcus sp.*
Eubacteria	Cyanophyta	Cyanophyceae	*Gloeocapsa sp.*
Eubacteria	Cyanophyta	Cyanophyceae	*Lyngbya aestuarii*
Eubacteria	Cyanophyta	Cyanophyceae	*Merismopedia elegans*
Eubacteria	Cyanophyta	Cyanophyceae	*Merismopedia glauca*
Eubacteria	Cyanophyta	Cyanophyceae	*Merismopedia punctata*
Eubacteria	Cyanophyta	Cyanophyceae	*Merismopedia quadruplicata*
Eubacteria	Cyanophyta	Cyanophyceae	*Merismopedia spp.*
Eubacteria	Cyanophyta	Cyanophyceae	*Microcystis sp. (incl. Anacystis)*
Eubacteria	Cyanophyta	Cyanophyceae	*Oscillatoria curviceps*
Eubacteria	Cyanophyta	Cyanophyceae	*Oscillatoria woronichini*
Eubacteria	Cyanophyta	Cyanophyceae	*Phormidium formosum*
Eubacteria	Cyanophyta	Cyanophyceae	*Phormidium limosum*
Eubacteria	Cyanophyta	Cyanophyceae	*Phormidium persicinum*
Eubacteria	Cyanophyta	Cyanophyceae	*Phormidium tergestinum*
Eubacteria	Cyanophyta	Cyanophyceae	*Planktothrix agardhii*
Eubacteria	Cyanophyta	Cyanophyceae	*Rhabdoderma lineare*
Eubacteria	Cyanophyta	Cyanophyceae	*Spirulina major*
Eubacteria	Cyanophyta	Cyanophyceae	*Spirulina subsalsa*
Eubacteria	Cyanophyta	Cyanophyceae	*Synechococcus spp. sensu lato*
Eubacteria	Cyanophyta	Cyanophyceae	*Trichodesmium sp.*

The other widely distributed marine cyanobacteria, *Prochlorococcus*, has, to our knowledge, not been reported in GoMA. *Prochlorococcus* commonly co-occurs with *Synechococcus* in subtropical and tropical open ocean waters, and is highly abundant in permanently stratified waters. However, *Prochlorococcus* is absent in subpolar and polar waters, and therefore its distribution range is limited to latitudes equatorward of approximately 45° [46]. The dominant source water in the Gulf of Maine is the Labrador Current and Scotian shelf, where *Prochlorococcus* does not occur [47].

### Aerobic anoxygenic phototrophs

Aerobic anoxygenic phototrophic (AAP) bacteria use bacteriochlorophyll in a photometabolism that does not split water to produce oxygen. It is assumed that organic matter or some other molecules act as electron donors. This bacterial photometabolism was previously known to occur in anaerobic environments but has now been shown to occur throughout the aerobic surface ocean [48], [49]. The AAP photometabolism occurs in a wide diversity of bacterial types [50], so the term AAP appears to best represent a functional group of bacteria rather than a phylogenetic group.

In a study conducted from 2001 to 2002, the abundance of AAP bacteria in GoMA ranged from a low of 7,600 cells ml^−1^ in March with a water temperature of 4.2°C, to a high of 98,400 cells ml^−1^ in October when the water was 11.7°C [51]. AAPs ranged from 1 to 9 % of the total bacteria in these samples, with higher proportions occurring during the warmer October sampling. The distribution of AAP bacteria generally followed that of phytoplankton, being more abundant in more productive waters. The emerging conclusion is that although AAP bacteria comprise only a small proportion of total oceanic bacterioplankton, the photoheterotrophic mode of the former is still more energetically efficient than the obligate hetetrophic mode of most bacterioplankton [52]. Indeed, AAP bacteria may have a high growth rate in the ocean [53].

### Proteorhodopsin bacteria

Proteorhodopsin is a retinal-binding protein that functions as a light-driven proton pump in many marine bacteria [54]. Variants of proteorhodopsins have been identified that show spectral adaptation to light quality in marine systems [55]. This photometabolism generates much less energy than photosynthesis, but possibly enough to give a growth advantage over obligate heterotrophs in the light [56].

The widespread occurrence of proteorhodopsin genes in metagenomic surveys [13], [57], [58] suggests that bacteria with a light-driven proton pump may be ubiquitous in pelagic environments. Profteorhodopsin genes found in GoMA by GOS are more than 80% dominated by variants adapted to green light. This distribution of variants appears common in northern coastal regions, but contrasts with the distribution in open ocean and tropical coastal waters where the blue variant dominates [13]. In a demonstration of whole genome sequencing of single cells isolated by cell sorting, Flavobacteria cells from GoMA in Boothbay Harbor waters were shown to contain proteorhodopsin genes [40].

## Eukaryotes

### Autotrophs

GoMA been characterized as a complex biogeographic transition area comprising species that are characteristic of several provinces [59]. For macroorganisms, the Gulf represents the northern distribution limit for many warm-water species and the southern distribution limit for many cold-water species. The microphytoplankton flora is essentially a mixture of temperate and boreal species, mostly diatoms, of both neritic and oceanic components.

Current and historical records of microalgae in GoMA document 665 names and 229 genera (Table 2, Table S1). The records include some algae generally considered freshwater algae but they were found in coastal, estuarine, and tidal river habitats that are considered part of the Gulf of Maine. Verification problems may arise in these records. There is often a lack of voucher material, drawings, photographs, or DNA, and these omissions impede modern verification of historical microalgal identities. For example, based upon electron microscopy and DNA sequences, the planktonic diatom commonly reported as *Skeletonema costatum*, as currently circumscribed [60] almost certainly does not occur in the Gulf, whereas the taxa in the Gulf are likely to belong to one of these three species: *Skeletonema grethae*, *S. menzelii*, *S. marinoi*. Additionally, nomenclatural problems arise because of name changes and newly discovered cryptic diversity found within old names. As a result of these problems, it is essentially impossible to use the historical data to assemble a fully accurate listing of marine microalgae. Nevertheless, microalgae remain the most broadly studied group of microbes in GoMA.

**Table 2:** Microalgae in the Gulf of Maine area (full list in Table S1).

Super Group	Division	Class	Number of scientific names
Chromalveolates	Alveolata	Dinophyceae	151
Chromalveolates	Cryptophyta	Cryptophyceae	4
Chromalveolates	Cryptophyta	Katablepharidophyceae	1
Chromalveolates	Haptophyta	Pavlovophyceae	1
Chromalveolates	Haptophyta	Prymnesiophyceae	31
Chromalveolates	Heterokontophyta	Bacillariophyceae	386
Chromalveolates	Heterokontophyta	Chrysophyceae	11
Chromalveolates	Heterokontophyta	Dictyochophyceae	6
Chromalveolates	Heterokontophyta	Eustigmatophyceaae	1
Chromalveolates	Heterokontophyta	Pelagophyceae	1
Chromalveolates	Heterokontophyta	Synurophyceae	1
Chromalveolates	Heterokontophyta	Xanthophyceae	1
Chromalveolates	Rhizaria	Cercozoa	2
Excavata	Euglenophyta	Euglenophyceae	6
Plantae	Charophyta	Zygnematophyceae	15
Plantae	Chlorophyta	Chlorophyceae	20
Plantae	Chlorophyta	Prasinophyceae	8
Plantae	Chlorophyta	Trebouxiophyceae	8
Plantae	Chlorophyta	Ulvophyceae	10
Plantae	Rhodophyta	Porphyridiophyceae	1

Diatoms (Bacillariophyta) are the most taxon rich group of algae in GoMA as they are elsewhere. A checklist of diatom species reported (and presumed native) from the Canadian and the neighbouring north, east, and west coasts of North America indicates approximately 893 valid names, representing 160 diatom genera that include 825 species and 69 subspecies taxa level [61]. A subset of these is found in GoMA, where approximately 100 genera and 345 species have been recorded, including 50 species of *Chaetoceros*, 34 species of *Navicula*, 23 species of *Nitzschia*, 15 species of *Coscinodiscus*, 13 species each of *Pleurosigma* and *Thalassiosira*, and 11 species of *Gyrosigma*. Almost certainly, these numbers underestimate the true diversity.

Dinoflagellates (Dinophyceae) have taxon records for 157 names and 26 genera. Of these, there are 47 species belonging to *Peridinium*, which is now generally regarded as a freshwater/brackish genus that has nearly 1000 named entities (but far fewer recognized by modern taxonomists). Other named genera in the historical records include *Alexandrium*, *Amphidinium*, *Ceratium*, *Cochlodinium*, *Dinophysis*, *Diplopsalis*, *Exuviella*, *Glenodinium*, *Gonyaulax*, *Gymnodinium*, *Gyrodinium*, *Heterocapsa*, *Katodinium*, *Mesoporos*, *Minuscula*, *Noctiluca*, *Obeliea*, *Oxyrrhis*, *Oxytoxum*, *Peridiniopsis*, *Phalacroma*, *Prorocentrum*, *Protoperidinium*, *Pyrophacus*, *Scrippsiella*, *Zygabikodinium*.

Haptophyta are a predominately marine group of algae, with only a very few species known from freshwaters. GoMA records include 34 names and 17 genera, which are mostly coccolithophores. Common genera include *Chrysochromulina*, *Diacronema*, *Emiliania huxleyi* ( = *Coccolithus huxleyi*, *Pontosphaera huxleyi*), *Pavlova*, and *Prymnesium*.

The green algal records include many marine species but also there are a number of freshwater taxa that have been reported from tidal rivers (e.g. *Pediastrum*, *Scenedesmus*). The class Prasinophyceae includes five marine genera represented by *Halosphaera viridia*, *Micromonas pusilla*, *Ostreococcus* sp., *Pycnococcus provasolii* and *Tetraselmis* spp.. The Trebouxiophyceae records consist of 6 genera: *Chlorella* spp., *Nannochloris* sp., *Chlorosarcinopsis halophilia*, *Oocystis minuta*, *Pyramimonas* spp., and *Schizochlamydella capsulata*.

Among the Chromalveolate microalgae, in addition to the diatoms and dinoflagellates mentioned above, there is good reprefsentation from a number of classes. The Cryptophyceae records include 4 genera (*Chilomonas marina*, *Chroomonas pauciplasta* (nom. nud.), *Hemiselmis rufescens*, and *Rhodomonas* spp.) but almost certainly many more cryptophytes occur in GoMA. Among the heterokont algae, there are a few known representatives for many classes. The silicoflagellate *Dictyocha speculum* and the related *Pseudopedinella* are two dictyochophytes, but it is likely that a number of others also occur in GoMA. For the Eustigmatophyceae, *Nannochloropsis granulata* has been identified using DNA sequences, but one assumes that many of the other *Nannochloropsis* species also inhabit this region. The Pelagophyceae, which generally occurs in the open oceans, has been found, as *Pelagococcus* spp. (apparently undescribed species), from the central regions of the Gulf. The Chrysophyceae and Synurophyceae, which are predominately freshwater groups, have been reported from tidal rivers and estuaries. Taxa include *Dinobryon*, *Synura* and *Uroglenopsis*. The Xanthophyceae, another predominately freshwater group, is represented by true marine species of *Vaucheria*, a filamentous alga that occurs in the benthos along the coastline. There are no records of Bolidophyceae, Aurearenophyceae, Raphidophyceae, and Synchromophyceae, all of which are mainly or entirely marine organisms. Amazingly, the fish-killing raphidophytes (*Chattonella*, *Heterosigma*) have never been reported even though there is intensive salmon fish farming in the Gulf.

A monitoring program initiated in 1987 in the southwest portion of the Bay of Fundy provides an ongoing record of morphotype diversity in the phytoplankton. To date, 55 species of dinoflagellates, 168 species of diatoms and several other species including flagellates have been observed in this area [62], [63], [64], [65], [66]. Records of the paralytic shellfish poisoning (PSP) producing organism, *A. fundyense*, have been further separated into its life cycle stages, which include: duplets or triplets (asexually dividing cells) that are observed early in the bloom, fusing (sexual reproduction where two cells fuse together), planozygotes (large cells formed from the fusing cells) and cysts or resting spores.

The nomenclature of the organism *A. fundyense* (formerly named *Gonyaulax*  =  *Protogonyaulax tamarense var excavatum*, *G. tamarense*) was revised by Balech in 1985 based on the apical plate structure and its lack of a ventral pore. There are 3 species of *Alexandrium* in the Gulf of Maine; *A. fundyense, A. tamarense* and more recently, *A. ostenfeldii*. Anderson et al. [67] provided this operational rule: “Two saxitoxin-producing species of *Alexandrium* occur in the Gulf of Maine: *A. fundyense* and *A. tamarense*. We consider these to be varieties of the same species [68]. Neither antibody nor oligonucleotide probes can distinguish between *A. fundyense* and *A. tamarense* from this region; only detailed analysis of the thecal plates on individual cells can provide this resolution. Since this is not practical for large numbers of field samples, for the purpose of this and other field studies, we use the name *A. fundyense* to refer to both forms”.

Seven species of Pseudo-nitzschia are known in the Bay of Fundy: *P. americana, P. delicatissima, P. pseudodelicatissima, P. fraudulenta, P. pungens, P. seriata and P. subpacifica.* Domoic acid produced by *P. pseudodelicatissima* may lead to amnesic shellfish poisoning (ASP). These species correlate differently with chemical and physical properties of seawater, suggesting that a multivariate approach may be a practical approach towards understanding the population dynamics of this group of related species [69].

In order to establish a baseline for species indigenous to the Bay of Fundy waters, a conservative approach is taken to list species that have been observed since 1995. From the 253 taxa identified since 1995, 8 dinoflagellate, 14 diatom and 5 additional taxa have been documented in the area for the first time. Those new species include the following: (dinoflagellates) *Alexandrium pseudogonyaulax*, *Amphidinium carterae*, *Amphidinium sphenoides*, *Ceratium macroceros*, *Polykrikos schwartzi, Preperidinium meunieri*, *Protoperidinium crassipes*, and *Pyrocystis lunata,* and (diatoms) *Attheya septentrionalis*, *Attheya longicornis*, *Chaetoceros radicans*, *Cylindrotheca gracilis*, *Grammxatophora serpentina*, *Lithodesmium undulatum*, *Mediopyxis helysia, Membraneis challengeri, Neodenticula seminae*, *Odontella sinensis*, *Proboscia eumorpha*, *Pseudo-nitzschia subpacifica*, *Pseudo-nitzschia fraudulenta* and *Thalassiosira punctigera* [70]. Most of the species new to the area are cold temperate species that tend to exist in many regions of the world with similar ecosystems to the Bay of Fundy. However, these species appear to have established populations in the Bay of Fundy as they have been observed during more than one year or annually since the time of first observance. Of the 27 new species observed in the area, the majority were observed in 2000 (9 species) and 2001 (14 species) whereas one new species was detected in each of the years 1997, 2002, 2004, and 2005.

### Heterotrophs and Mixotrophs

On the basis of morphotypes, heterotrophic eukaryote species richness appears to be low. Named or unnamed, there are 9 species of aloricate ciliates, 24 species of loricate ciliates, and 1 species of heterotrophic dinoflagellate (Table 3).

**Table 3:** Heterotrophic protists in the Gulf of Maine area.

Group	Species	Location	Reference
Aloricate ciliates	*Didinium sp.*	Halifax Harbour	Gifford 1988
Aloricate ciliates	*Laboea sp.*	Isles of Shoals	Montagnes et al. 1988
Aloricate ciliates		Bay of Fundy	Martin et al. 2006
Aloricate ciliates	*Laboea strobila*	Damariscotta estuary	Saunders 1995
Aloricate ciliates		Georges Bank	Stoecker et al. 1989
Aloricate ciliates	*Lohmaniella sp.*	Isles of Shoals	Montagnes et al. 1988
Aloricate ciliates	*Myrionecta rubra (ex Mesodinium rubrum)*	Damariscotta estuary	Saunders 1995
Aloricate ciliates		Halifax Harbour	Gifford 1988
Aloricate ciliates		Georges Bank	Stoecker et al. 1989
Aloricate ciliates		Bay of Fundy	Martin et al. 2006
Aloricate ciliates	*Strobilidium spp.*	Isles of Shoals	Montagnes et al. 1988
Aloricate ciliates		Bay of Fundy	Martin et al. 2006
Aloricate ciliates	*Strombidinopsis sp.*	Isles of Shoals	Montagnes et al. 1988
Aloricate ciliates	*Strombidium spp.*	Isles of Shoals	Montagnes et al. 1988
Aloricate ciliates		Georges Bank	Stoecker et al. 1989
Aloricate ciliates		Bay of Fundy	Martin et al. 2006
Aloricate ciliates	*Tontonia spp.*	Isles of Shoals	Montagnes et al. 1988
		Georges Bank	Stoecker et al. 1989
Loricate ciliates	*Eutintinnus apertus*	Damariscotta estuary	Saunders 1987
Loricate ciliates	*Eutintinnus pectinis*	Damariscotta estuary	Saunders 1987
Loricate ciliates	*Eutintinnus sp.*	Bay of Fundy	Martin et al. 2006
Loricate ciliates	*Favella sp.*	Damariscotta estuary	Saunders 1987
Loricate ciliates		Bay of Fundy	Martin et al. 2006
Loricate ciliates	*Helicostomella subulata*	Damariscotta estuary	Saunders 1987
Loricate ciliates	*Helicostomella spp.*	Bay of Fundy	Martin et al. 2006
Loricate ciliates	*Parafavella denticulata*	Damariscotta estuary	Saunders 1987
Loricate ciliates	*Parafavella parumdentata*	Damariscotta estuary	Saunders 1987
Loricate ciliates	*Parafavella spp.*	Bay of Fundy	Martin et al. 2006
Loricate ciliates	*Ptychocylis obtusa*	Damariscotta estuary	Saunders 1987
Loricate ciliates	*Ptychocylis spp.*	Bay of Fundy	Martin et al. 2006
Loricate ciliates	*Stenosomella olivia*	Damariscotta estuary	Saunders 1987
Loricate ciliates	*Stenosomella steini*	Damariscotta estuary	Saunders 1987
Loricate ciliates	*Tintinnidium fluviatile*	Damariscotta estuary	Saunders 1987
Loricate ciliates	*Tintinnopsis acuminata*	Damariscotta estuary	Saunders 1987
Loricate ciliates	*Tintinnopsis baltica*	Damariscotta estuary	Saunders 1987
Loricate ciliates	*Tintinnopsis campanula*	Damariscotta estuary	Saunders 1987
Loricate ciliates		Bay of Fundy	Martin et al. 2006
Loricate ciliates	*Tntinnopsis kofoidi*	Damariscotta estuary	Saunders 1987
Loricate ciliates	*Tintinnopsis levigata*	Damariscotta estuary	Saunders 1987
Loricate ciliates	*Tintinnopsis lobiancoi*	Damariscotta estuary	Saunders 1987
Loricate ciliates	*Tintinnopsis minuta*	Damariscotta estuary	Saunders 1987
Loricate ciliates	*Tintinnopsis nucula*	Damariscotta estuary	Saunders 1987
Loricate ciliates	*Tintinnopsis rapa*	Damariscotta estuary	Saunders 1987
Loricate ciliates	*Tintinnopsis tubulosoides*	Damariscotta estuary	Saunders 1987
Heterotrophic dinoflagellate	*Gymnodinium sp.*	Halifax Harbour	Gifford 1988

Eukaryotic heterotrophs have not been monitored systematically in any region of GoMA. The most intensive sampling was performed in 3 areas for limited periods of time. Montagnes et al. [71] focused exclusively on planktonic cilates at 3 stations of about 20 m depth off the Isles of Shoals over 15 months during 1985–1986. Morphological species were identified for the purpose of estimating total ciliate biomass at each station. Thirty-four morphospecies were identified, with abundances dominated by the genera *Strombidium, Lohmaniella, Laboea, Tontonia, Strobilidium* and *Strombidinopsis*.

Gifford and Sieracki (unpublished) sampled nano- and microheterotrophs throughout the water column in 3 hydrographically distinct areas of Georges Bank during January-June, 1995. Due to preservation with iodine-based fixative, which obscures internal detail, morphotypes of ciliates and dinoflagellates were only identified and subdivided into size classes. The number of morphotypes varied over season. The numerical abundances of all taxa were lowest in winter and early spring and increased following the spring bloom as the water column warmed. Forms larger than 20 µm were more abundant during winter and spring when the water column was fully mixed and forms smaller than 20 µm were more abundant during summer after the onset of stratification.

Stoecker et al. [72] mapped patterns of ciliate abundance along three transects on Georges Bank in July 1987. Total numerical abundances ranged from 600–13,000 cells l^−1^ and were highest on the shallow crest of the Bank. The mixotroph, *Myrionecta rubra* (ex *Mesodinium rubra*), contributed ca. 30% of total ciliate numbers on average, and mixotrophic oligotrich ciliates comprised ca. 34% of the ciliate fauna. The study included one station located in the Gulf of Maine in Georges Basin, where ciliates were less abundant than on the Bank.

In a 14-month study devoted exclusively to tintinnid ciliates in the Damariscotta River estuary, Saunders [73] identified 20 species, 10 of which were of the genus *Tintinnopsis*. Seasonal variation of tintinnid numbers (ranging from ca. 10 to several thousand cells l^−1^) tracked water temperature and chlorophyll, with numerical maxima in spring and summer and a minimum in winter. Other ciliates, primarily oligotrichs, were usually more abundant by an order of magnitude. The mixotrophic ciliate *Laboea strobila* was present year-round in relatively low abundance (ca. 2–40 cells l^−1^). Another mixotroph, *Myrionecta rubrum*, was present and abundant (ca. 1,000–40,000 cells l^−1^) only during winter and spring. Because of its relatively large size and high numerical abundance, it contributed significantly to the total ciliate standing stock when present [74]. In this estuary, the predator-prey link between protists and their presumptive food source is evident in lagged annual maxima [75].

In the deeper waters of the Gulf of Maine, protists can be found at high abundances (up to 100 cells l^−1^) at depths from 55 to 100 m, which is well below the euphotic zone, pycnocline, and the depths of peak biomass and production of both phytoplankton and bacteria [76]. Here, the most abundant protists were the heliozoan *Sticholonche* sp. and various tintinnids, principally the genus *Tintinnopsis*. These populations may derive their nutrition from particulates which settle out of surface waters rather than from local phytoplankton or microbial production.

## Inventory

For each selected microbial group *k*, a GoMA-wide inventory N*_k_* can be made as follows. In each physiographic region *j* (Fig. 1), total standing abundance *N_kj_* is computed as the sum over all *m* depth layers *z_i_* of the product of two functions, namely the depth-dependent variation of GoMA-wide cell density *n_k_*(*z_i_*) and the depth-dependent variation of water volume in that region *v_j_*(*z_i_*).



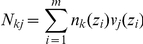



**Figure 2:**
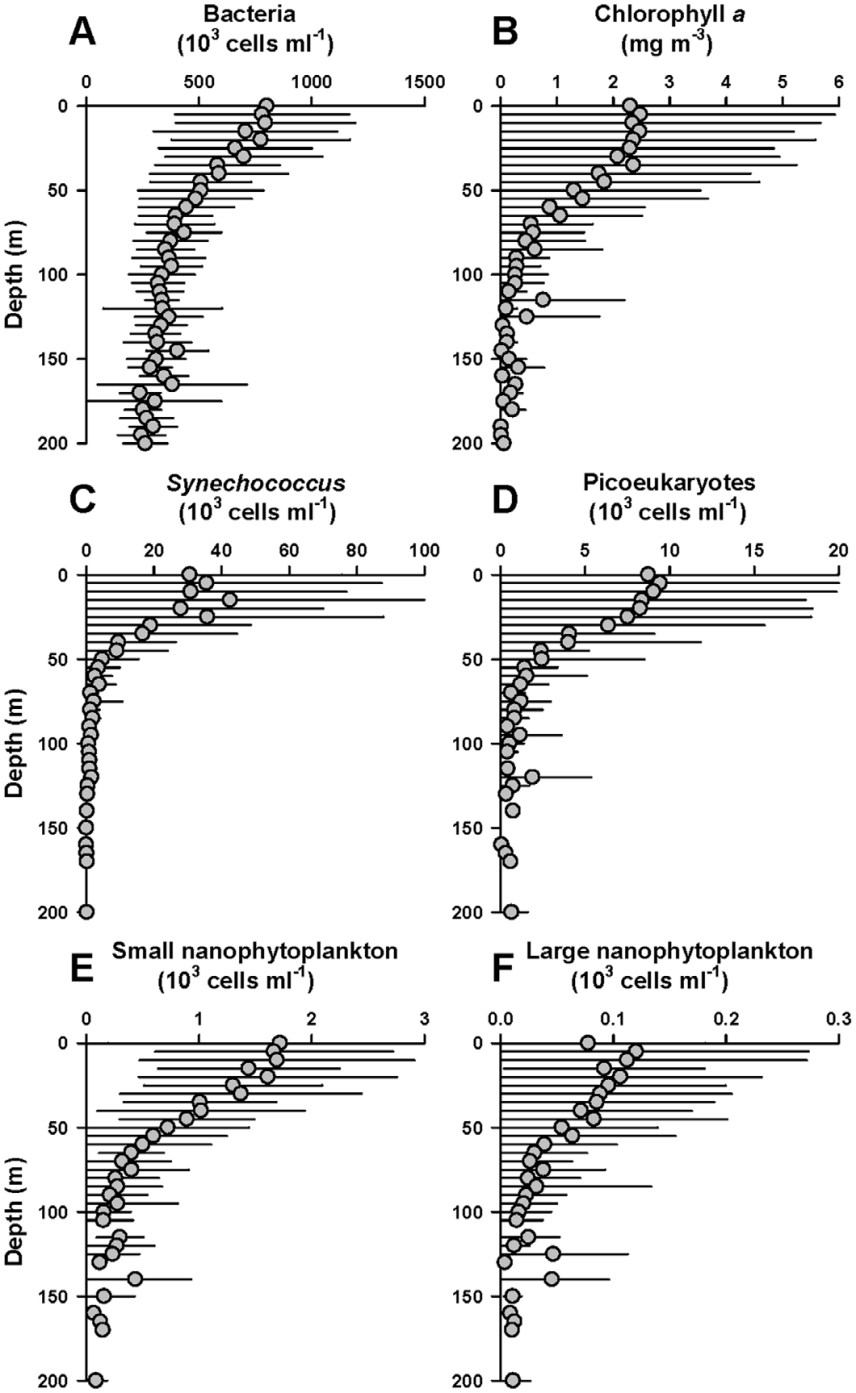
Abundance of microbial groups on the Scotian Shelf. (A) Bacteria (prokaryotes excluding cyanobacteria); (B) Chlorophyll a; (C) *Synechococcus;* (D) Picoeukaryotic phytoplankton; (E) Small nanophytoplankton; (F) Large nanophytoplankton. Data are from the Atlantic Zone Monitoring Program and binned into 5 m depth intervals. Profiles indicate average values and standard deviations from a network of stations on the Scotian Shelf sampled mainly in the spring and autumn from 1997 to 2010.

**Figure 3:**
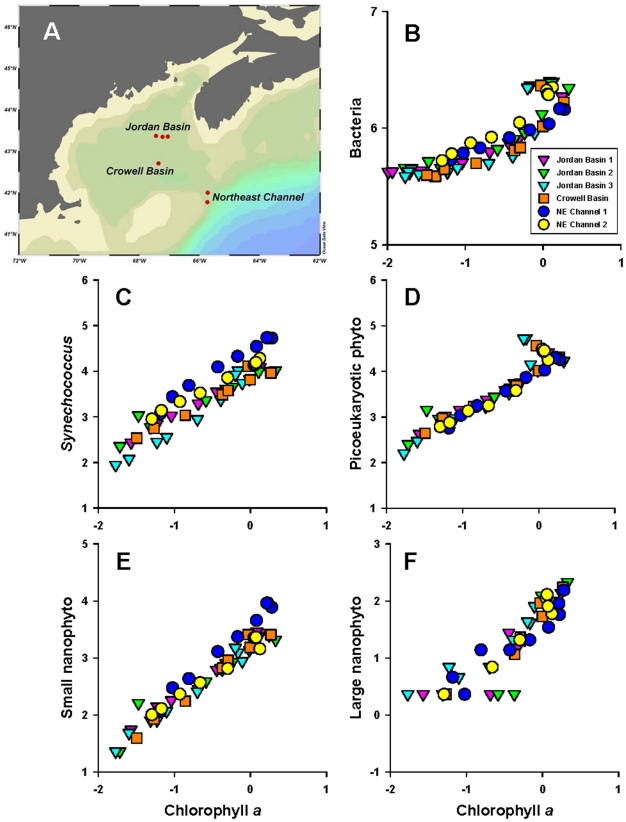
Depth-varying correlations of microbial abundance versus chlorophyll *a* concentration in the Gulf of Maine. (A) Map of 3 stations in Jordan Basin, 1 station in Crowell Basin, and 2 stations in the Northeast Channel. Samples were collected in the upper 200 m from June 13–16, 2005 during the Discovery Corridor cruise. (B) Bacteria; (C) *Synechococcus;* (D) Picoeukaryotic phytoplankton; (E) Small nanophytoplankton; (F) Large nanophytoplankton. Microbial abundance in units of log cells ml-1; chlorophyll *a* concentration in units of log mg m^-3^.

For this calculation, depth-dependent variations of cell density (cells m^−3^) can be parameterized from polynomial curve fits to GoMA-wide depth-binned average measurements. Such measurements (Fig. 2) are available only for the following 5 nominal microbial groups: bacterioplankton (prokaryotes excluding cyanobacteria), *Synechococcus* spp., picoeukaryotic phytoplankton, small nanophytoplankton (2–10 µm), and large nanophytoplankton (10–20 µm). The abundance of these cells is measured by flow cytometry [45] from full depth hydrographic casts across the entire Scotian Shelf and Slope in a network of sampling stations comprising the Atlantic Zone Monitoring Program [42] and at selected hydrographic stations in the Gulf of Maine proper. These profiles (Fig. 2) are taken to represent the vertical distribution of the microbes across the entire GoMA. Strong correlations are evident in the depth varying distributions of each microbial group with total phytoplankton biomass (indicated by chlorophyll *a* concentration) (Figs. 2,3).

Depth-dependent variations of water volume (m^3^) can be derived from hypsometric analysis (Text S1). For calculation, each water layer *i* is set to 10 m thickness. The gulf-wide inventory is the sum of region-specific standing stocks over all 14 physiographic regions.
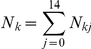



In GoMA, a provisional estimate for the minimum abundance of cell-based microbes is 1.7×10^25^ organisms. The component inventories are 1.6×10^25^ bacterioplankton, 2.7×10^23^ *Synechococcus*, 8.1×10^22^ picoeukaryotic phytoplankton, 1.7×10^22^ small nanophytoplanton, and 1.2×10^21^ large nanophytoplankton (Table 4). In the same order, the ratios are 13,055 : 220 : 65 : 14 : 1. This provisional estimate does not account for viruses, nor any of the heterotrophic eukaryotes or larger phototrophic eukaryotes. Because of allometric constraints, the larger organisms exist at lower cell densities and would not add substantially to the total numerical inventory.

**Table 4:** Microbial cell inventory (number of cells) in the Gulf of Maine area partitioned by physiographic region as defined in Supporting Information (Text S1).

Region	Bacterioplankton	*Synechococcus*	Picoeuk-phyto	Small Nanophyto	Large Nanophyto
Scotian Coastal Shelf	2.08E+23	6.37E+21	1.84E+21	3.78E+20	2.64E+19
Scotian Shelf	1.85E+24	3.60E+22	1.08E+22	2.35E+21	1.70E+20
Browns Bank	1.46E+23	3.76E+21	1.12E+21	2.41E+20	1.73E+19
Eastern Coastal Shelf	2.81E+23	8.50E+21	2.46E+21	5.05E+20	3.53E+19
Bay of Fundy	4.95E+23	1.36E+22	3.95E+21	8.20E+20	5.77E+19
Northern Coastal Shelf	4.88E+23	1.49E+22	4.31E+21	8.81E+20	6.15E+19
Southern Coastal Shelf	2.74E+23	8.77E+21	2.51E+21	5.07E+20	3.52E+19
Georges Bank	1.86E+24	5.17E+22	1.51E+22	3.17E+21	2.25E+20
Georges Basin	4.14E+23	5.31E+21	1.59E+21	3.48E+20	2.51E+19
Jordan Basin	5.66E+23	8.64E+21	2.59E+21	5.66E+20	4.09E+19
Wilkinson Basin	6.08E+23	9.14E+21	2.74E+21	5.99E+20	4.32E+19
Central Gulf of Maine	4.29E+24	7.62E+22	2.29E+22	4.99E+21	3.60E+20
Continental Slope	4.32E+24	2.82E+22	8.46E+21	1.85E+21	1.33E+20
Bear Seamount	4.08E+23	2.12E+21	6.34E+20	1.39E+20	1.00E+19
**TOTAL**	**1.62E+25**	**2.73E+23**	**8.09E+22**	**1.73E+22**	**1.24E+21**

Cell-based microbes, here as anywhere else in the ocean, are overwhelmingly dominated by prokaryotes in number. We may expect the number of viruses to be about ten-fold greater. The estimate of global ocean prokaryote abundance is 1.2×10^29^ [77], of which 0.014% can be ascribed to GoMA. The largest contributing regions are the continental slope (which is deep) and the central Gulf of Maine (which is areally extensive). About 26% of the prokaryote inventory is found in each of these two regions. Georges Bank and the western Scotian Shelf each contribute about 11% of the prokaryote inventory. Georges Bank holds less water volume than the western Scotian Shelf, but the water on the Bank is better illuminated because of shallower mean depth. All other regions combine to contribute 24%, but none exceeds 4% individually.

For bacterioplankton, biomass can be estimated from cell abundance using a conversion of 24 fg dry weight per cell [78]. In the 9 physiographic regions constituting 122,498 km^2^ of surface area in the Gulf of Maine proper (Browns Bank, Eastern Coastal Shelf, Bay of Fundy, Northern Coastal Shelf, Southern Coastal Shelf, Georges Basin, Jordan Basin, Wilkinson Basin, and Central Gulf of Maine – Table S1), the biomass equivalent of the sum of 7.56×10^24^ bacteria is 1.81×10^5^ tons dry weight. Therefore the areal concentration of bacterioplankton biomass in the Gulf of Maine proper is about 1.48 tons dry weight per square kilometer. This empirical estimate is only 27% of the value used to satisfy trophic demand in one ecosystem model [6] and may prompt a need for sensitivity analysis of the energy budget in this ecosystem.

## Richness

The estimation of microbial richness is fraught with difficulties in all aspects: conceptual, theoretical, statistical, empirical, and validational [79]. Indeed, the probable irrelevance of the species concept for prokaryotes [80] means that there is no sensible answer, at least in conventional terms familiar to those who study multicellular eukaryotes. We do not dwell on these discussions in spite of their importance. Instead, we use scaling relationships to estimate taxonomic richness of prokaryotes and phytoplankton in operational units for the comparative purpose of placing GoMA in a global context.

Taxonomic richness can be estimated from the taxon-abundance distribution using two measured variables [81]: the total number of individuals in the community (N*_T_*) and the number of individuals comprising the most abundant members of that community (N*_max_*). For prokaryotes, N*_T_* in GoMA is 1.6×10^25^ (Table 4) and the most common member is the SAR11 clade *Pelagibacter ubique* whose abundance may be taken to be about 25% of the total [82]. For phytoplankton, N*_T_* in GoMA is 3.7×10^23^ (Table 4) and the member with the largest number of individuals is *Synechococcus*, with N*_max_* 2.7×10^23^ (Table 4). Using the nomogram relating taxonomic richness to N*_T_* for different values of the ratio N*_T_* /N*_max_* [81], we estimate that GoMA could have between 10^5^ and 10^6^ different taxa of prokaryotes, and between 10^3^ and 10^4^ different taxa of phytoplankton belonging to all size classes. These are provisional estimates of richness, based on untested simplifying assumptions and derived from provisional estimates of microbial inventories.

A more constrained approach to estimating richness is the taxa-area relationship based on empirical measurements. The assembly of local communities from a metacommunity leads to the idea that ecosystem size sets an upper bound to the achievable diversity of taxa. Local communities typically are subsets of regional taxa pools, and the subsets are reduced in ecosystems of smaller size. For phytoplankton, a power law scales species richness (*S*) to ecosystem surface area (*A*) across more than 15 orders of magnitude in spatial extent: *S* = 62.9*A* ^0.134^ [83]. By inference, GoMA may have 328 distinct phytoplankton morphospecies in the entirety of its 221,990 km^2^. This contrasts with the 665 taxonomic names identified by actual observation (Table 3). At the smaller scale of one particular physiographic region, it is notable that the same scaling relationship estimates 223 phytoplankton species in the Bay of Fundy (12,544 km^2^) which is very close to the 253 morphotaxa recognized by actual microscopic analysis.

In GoMA, it seems that morphological diversity in microplankton is well-described but the true extent of taxonomic diversity, especially in the femtoplankton, picoplankton and nanoplankton – whether autotrophic, heterotrophic, or mixotrophic, is unknown.

## Supporting Information

Text S1 Definition of physioregions in GoMA with a summary of area, volume, and mean depth for physioregions.
(PDF)

Table S1 List of microalgae and cyanobacteria in GoMA.
(XLS)
