# Supplementary material for: Planktonic Microbes in the Gulf of Maine Area
Source: PLoS One. 2011 Jun 15;6(6):e20981. doi: 10.1371/journal.pone.0020981 (PMC3115965; doi:10.1371/journal.pone.0020981)
Supplement: Text S1 — Definition of physioregions in GoMA with a summary of area, volume, and mean depth for physioregions. (PDF) [file pone.0020981.s001.pdf]

SUPPORTING INFORMATION

Planktonic microbes in the Gulf of Maine area

William K.W. Li[1*]
Robert A. Andersen[2]
Dian J. Gifford[3]
Lewis S. Incze[4]
Jennifer L. Martin[5]
Cynthia H. Pilskaln[6]
Juliette N. Rooney-Varga[7]
Michael E. Sieracki[2]
William H. Wilson[2]
Nicholas H. Wolff[4]

---

[1] Fisheries and Oceans Canada, Bedford Institute of Oceanography, Dartmouth, NS, B2Y 4A2, Canada
[2] Bigelow Laboratory for Ocean Sciences, West Boothbay Harbor, ME 04575, USA
[3] Graduate School of Oceanography, University of Rhode Island, Narragansett, RI 02882, USA
[4] Aquatic Systems Group, University of Southern Maine, Portland, ME 04101, USA
[5] Fisheries and Oceans Canada, Biological Station, St Andrews, NB, E5B 2L9 Canada
[6] School for Marine Science and Technology, University of Massachusetts, North Dartmouth, MA 02747, USA
[7] Department of Biological Sciences, University of Massachusetts Lowell, Lowell, MA 01854, USA

* To whom correspondence should be addressed: Email:Bill.Li@dfo-mpo.gc.ca

Definition of physiographic regions in the Gulf of Maine Area

L. Incze, N. Wolff and A. Adamek defined physioregions for the Gulf of Maine Area and calculated the area and volume for each. Digital Bathymetry was obtained from the US Geological Survey (see Open-file Report 98-801). The eastern portions of the Scotian Shelf and Continental Slope (2% of the GoMA study area) extend beyond the available data in this dataset and are not included in these calculations. The selection of physioregions and delineation of their boundaries inevitably involved some subjective decisions.

Some of the delineations followed smoothed isobaths constructed from the bathymetric data using Geographical Information System software (Esri). The coastal shelves and Browns Bank were defined as ≤100 m. The seaward boundaries of Georges Bank and the Scotian Shelf were drawn at the 200 m isobaths, and the mouth of the Northeast Channel (Central Gulf of Maine) was defined by a slightly curved line along the outside of its sill, although the sill is at all points deeper than 200 m. This was a definition of convenience to define a coherent shelf. The portion of the Continental Slope calculated in this study extends from these outer shelf boundaries to 2,000 m, or about mid-slope. The eastern end of the GoMA study area is a research and monitoring line (Halifax Line) used in the Canadian Atlantic Zone Monitoring Program (AZMP). We used another AZMP transect (Browns Bank Line) to define the boundary between the western Scotian Shelf (both coastal and outer portions) and the Gulf of Maine. The line extends from Cape Sable, Nova Scotia to the intersection of the Continental Slope with the eastern edge of the Northeast Channel boundary. The southwestern edge of the study area was defined from southeastern Cape Cod around Nantucket Shoals along a smoothed line approximating the western edge of the Great South Channel (no single depth was practical to use for this purpose), and finally south along 69° 20′ W longitude to the 2,000 m isobath.

In the interior of the Gulf of Maine, we defined the Bay of Fundy by a straight line between West Quoddy Head, Maine and Cape St. Mary, Nova Scotia. The boundary between Georges Bank/Great South Channel and the Southern Coastal Shelf was located at the western edge of the submarine valley which leads southward into the Great South Channel . The northern boundary of Georges Bank was defined by the 100 m isobath, and a transition to the 200 m isobath at the seaward edge of the bank occurs along the edge of the Northeast Channel . In the Central Gulf of Maine, we distinguished between the three deep basins and the rest of the central Gulf, defining the boundaries of Jordan Basin and Wilkinson Basin at 200 m, and the shoulders of Georges Basin, which lies in a deeper portion of the Gulf, at 250 m. Georges Basin contains the deepest place (-366 m) on the shelf in the study area. The Bear Seamount physioregion encompasses the seamount and some of the surrounding lower slope, reaching a depth of 3,000 m.

Summary of area, volume, and mean depth for physioregions in the Gulf of Maine Area.

Region/Physioregion	Area (km^2)	Volume (km^3)	Mean Depth (m)
Coastal Shelves (≤100 m deep)			
Scotian Coastal Shelf	6,206.5	353.1	-52
Eastern Coastal Shelf	7,760.0	481.1	-57
Northern Coastal Shelf	14,115.9	832.3	-54
Southern Coastal Shelf	8,203.4	457.4	-51
Bay of Fundy	12,544.1	919.9	-68
Open Shelves			
Scotian Shelf	27,868.7*	4,178.2	-144
Central Gulf of Maine**	59,041.0**	10,357.1**	-170**
Major Basins (Gulf of Maine)			
Georges Basin	4,110.5	1,246.0	-298
Jordan Basin	6,694.2	1,523.7	-222
Wilkinson Basin	7,077.9	1,654.9	-228
Offshore Banks			
Browns Bank	2,951.4	268.4	-85
Georges Bank	41,933.8	3,353.5	-75
Continental Slope (to 2000 m)	21,845.2***	26,128.1	-1,191
Bear Seamount (and surrounding slope)	1,637.5	3,937.6	-2,400

Footnotes:

* The total area, including unshaded section in Figure 1, is 29,928.9 km^2.

** This calculation does not include the three major basins detailed in the next subsection. Areas and volumes for the basins can be added to the Central Gulf of Maine data to obtain a grand total for that physioregion.

*** The total area, including the unshaded section in Figure 1, is 24,325.4 km^2.
